# Development of combined hypersonic test facility for aerothermodynamic testing

**DOI:** 10.1371/journal.pone.0298113

**Published:** 2024-02-14

**Authors:** Sungmo Yang, Ilsung Choi, Gisu Park

**Affiliations:** Department of Aerospace Engineering, Korea Advanced Institute of Science and Technology, Daejeon, Republic of Korea; Tianjin University, CHINA

## Abstract

In this study, a combined test facility was developed using a combination of an arc-jet tunnel and a shock tunnel for aerothermodynamic testing. The performance validation of individual parts was performed, and results were obtained from the combined test. A small-scale Huels-type arc-jet tunnel was used to preheat the test model by aerodynamic heating before conducting the experiments in the shock tunnel to duplicate the hot surfaces of flight objects encountered during hypersonic flight. The high-enthalpy flow in the arc-jet tunnel provided a heat flux of 1.99±0.03 MW/m^2^ for a flat-faced model of 10 mm diameters, and the flow condition of the shock tunnel used in this study simulated a Mach 5 flight at a pressure altitude of about 24 km. The two combined experiments employing different shape and material models were carried out to examine the effect of aerothermodynamic phenomena. In the first experiment, the effect of ablation-induced shape change on the fluid-structure was investigated using a cone model manufactured of AL6061 material. The effect of surface roughness on the fluid-structure was examined in the second experiment, which used a hemisphere model constructed of STS303 material. Although substantial findings could not be validated due to the limits of qualitative evaluations utilizing visualization methods, however preheating-related changes in surface roughness were found. As a follow-up study, a force measuring experiment based on the test procedures is being carried out at this facility utilizing a preheated model with an accelerometer. The performance and experimental results obtained using this integrated setup are discussed in detail, highlighting the potential of this combined hypersonic test facility.

## Introduction

Strong shock waves formed around actual hypersonic flight objects, such as space debris, re-entry capsules, and hypersonic vehicles, behave differently in perfect gases. Structural heat loads generated by high-enthalpy flow are critical properties of these objects. When a hypersonic flow develops, the thermochemical phenomenon changes the flow behavior and heat transfer distribution around an object while its surface is heated. Hot surfaces of hypersonic flight objects exhibit several aerothermodynamic properties. Consequently, it influences the thermochemical phenomena involving flow behavior, such as thermochemical nonequilibrium phenomena [1]. A thorough consideration of the aerothermodynamic phenomenon of hypersonic flow is required to develop hypersonic vehicles. Therefore, a hypersonic ground test facility needs to simulate hot-surface aerothermodynamics to better design and build hypersonic vehicles.

Ground-based tests, numerical simulations, and flight tests complement the research, development, testing, and evaluation (RDT&E) of hypersonic vehicles. Research using ground-based test facilities is primarily conducted in the early stages of the RDT&E of hypersonic vehicles to predict and simulate the real gas effect of hypersonic flow on the performance of a vehicle, such as force, moment, and pressure/thermal distribution [2]. Data obtained using ground experiments can be used as properties for computational fluid dynamics (CFD) modeling and can provide validation data. In addition, flight tests are mainly performed in the final stage of RDT&E using verified models established from ground-based tests and CFD because of their high costs and risks. The parameters that can be simulated using a ground-based test facility include the freestream Mach number, freestream Reynolds number, freestream velocity, temperature, pressure, total enthalpy, and thermochemical properties [3]. Three types of high-enthalpy facilities widely used for simulating hypersonic flows are 1) blow-down wind tunnels, 2) plasma wind tunnels, and 3) impulse-type facilities. Blow-down wind tunnels have the advantage of long testing periods; however, cold hypersonic flow phenomena occur in this low-enthalpy facility owing to their low total temperature. These limitations can be mitigated using additional heating devices in the reservoir; however, technological limits exist for continuously working heaters. A Mach number simulation is somewhat limited; however, it cannot simulate the high Reynolds number in the flight environment of a hypersonic flight vehicle [4]. Plasma wind tunnels, such as arc-jet tunnels, have the advantage of being able to simulate extremely high-enthalpy flow and extended operation times; therefore, they are often used in radiation, ablation, gas-surface interactions, and thermal protection system studies. However, the arc-jet facility permits the testing of only a few aerothermodynamic properties, and it is somewhat limited to simultaneously match all flight parameters [5]. Impulse-type facilities such as shock tubes/tunnels can yield uniform and steady high-enthalpy flows that simulate representative flow conditions (pressure, temperature, and enthalpy) under target flight conditions. They are mainly used for analyzing flow characteristics around hypersonic flight objects as well as in radiation and nonequilibrium studies. However, the surface temperature of the test model cannot be increased to the actual temperature of hypersonic objects by aerodynamic heating in actual flights owing to the short test period (typically in milliseconds). Therefore, hot-surface aerothermodynamic phenomena cannot be simulated during testing. Various ground-based facilities are used to investigate these phenomena due to the inability of a single facility to replicate a hypersonic environment [6].

**Fig 1:**
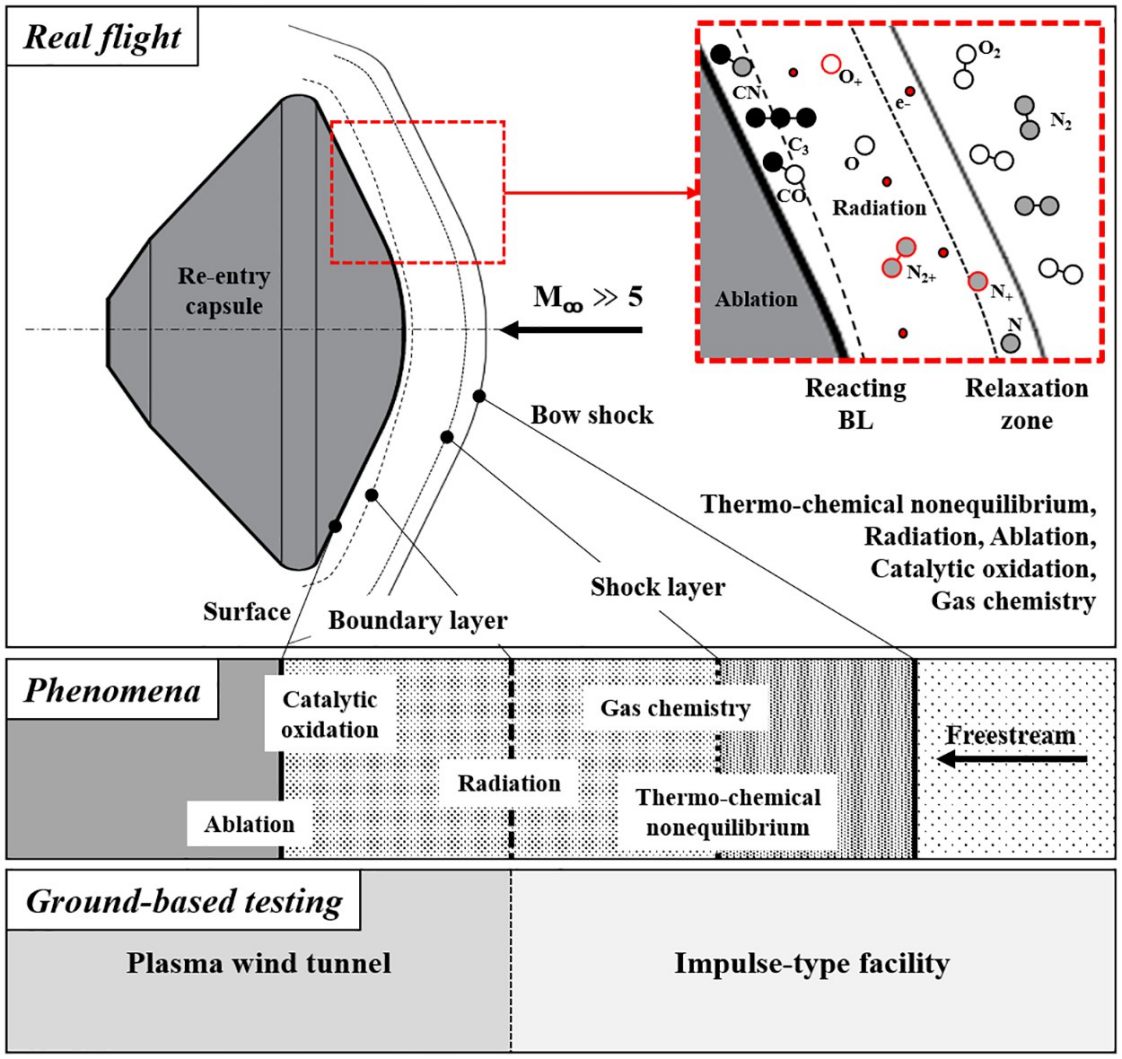
Typical aerothermodynamic phenomena and associated ground-based test facilities for simulation.

Chazot, et. al. [7] provide a detailed and comprehensive description of the highly complex flow phenomena surrounding hypersonic objects and the high-enthalpy facilities and plasma wind tunnels used to investigate these phenomena. Fig 1 shows the aerothermodynamic phenomena and associated ground-based facilities for simulating hypersonic flows. This figure illustrates the strong shock waves that occur around hypersonic flight objects and phenomena such as thermochemical nonequilibrium, radiation, ablation, catalytic oxidation, gas chemistry, and others that are due to aero-thermal effects. Strong shock waves around hypersonic flight objects intensify thermochemical nonequilibrium effects. When the gases around the vehicle encounter the shock wave, the gas molecules are rapidly compressed and heated, causing dissociation and ionization reactions to occur. The high temperature and pressure behind the shock promote the formation of highly reactive species such as free radicals and excited atoms. These species contribute to the nonequilibrium gas chemistry of the flow field. Thermochemical nonequilibrium caused by aerodynamic effects has a significant impact on the performance of hypersonic vehicles. It influences many aspects, including heat transfer properties, surface chemistry, and flow characteristics around the object. The nonequilibrium distribution of species changes the rate of heat transfer between the object surface and the surrounding gas, resulting in uneven surface heating and different temperature profiles. This, in turn, can affect the aerodynamic forces acting on the object, such as lift and drag [8, 9].

**Fig 2:**
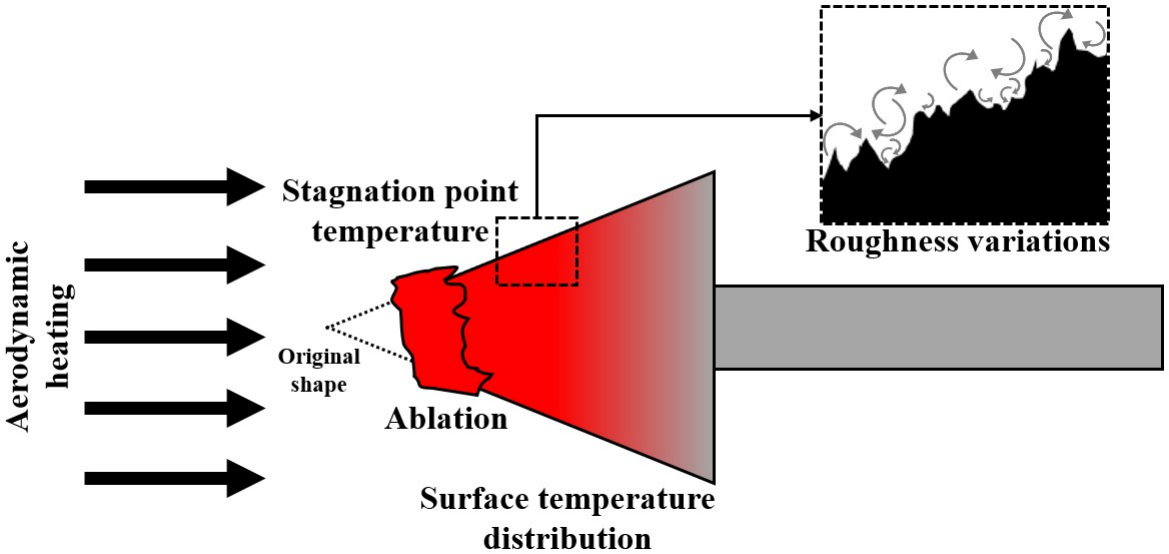
Simulatable aerothermodynamic phenomena using aerodynamic heating method.

As mentioned earlier, impulse-type facilities can generate high-pressure and enthalpy flows that match the target flight conditions. However, the surface temperature of the model barely increases during the experiment, which is a limitation for experiments using impulse-type facilities. Previous work successfully overcame the challenges of applying different force measurement techniques in short test times in a shock tunnel, but it was not possible to simulate the heating of the model surface temperature due to equipment limitations [10]. Therefore, various experimental techniques and ground test equipment concepts are being developed to overcome the limitations of impulse-type facilities. Hirschel [11] proposed a “hot experimental technique” for researching hot-surface aerothermodynamic phenomena that overcome the limitations of hypersonic test facilities. Since then, studies on hot-surface aerothermodynamic phenomena have been conducted in impulse-type facilities using preheated models based on a few preheating methods. The most widely used preheating method is the electrical resistive heating device placed inside the model to rapidly increase the wall temperature to the target temperature without disturbing the external test flow, used by research groups at the University of New South Wales, RWTH Aachen University, and German Aerospace Center (DLR) [12–28]. The S1 Table contains a detailed summary of the preheating techniques used in impulse-type facilities. The University of Queensland established a test model preheating method using a direct current (DC) source and high-resistance materials called the high-electrical resistive heating method [29]. However, this method has the disadvantage that it is difficult to apply the energy source to the highest thermal load locations such as stagnation points or leading edges experienced in real flights. Using a plasma facility to heat a test model is a promising alternative to overcome the limitations of using electrical heaters to simulate the heating experienced by hypersonic objects owing to flow. Fig 2 shows the aerothermodynamic phenomena that can be simulated by using the aerodynamic heating method proposed in this paper. This method has the advantage of more accurately simulating real-world conditions. These conditions include factors such as stagnation point temperature, ablation, effects of roughness variations, and surface temperature distribution.

Previous conceptual studies have proposed using plasma facilities to heat models and impulse-type facilities to perform experiments. The initial concept was proposed in a paper from the Federal Military University of München, describing the development of a new piston-driven shock tunnel (HELM). The author states that due to the short test time in the shock tunnel, the thermal load on the test model is only a small degree, resulting in thermal boundary layer conditions that differ from the real world. The proposed method involves integrating a plasma-wind tunnel perpendicular to the flow direction of the piston-driven shock tunnel to heat the model with the plasma heated flow. The model is then rotated 90° either before or during the establishment of flow in the shock tunnel nozzle to subject it to the test flow. The author suggested that this technique could be implemented once the necessary infrastructure is in place. However, since the publication of this paper, there have been no reported advancements or official outcomes [30]. Subsequently, a similar conceptual study was published by the Oxford researchers. Conceptual design involves a dual facility setup using an arc-jet tunnel as a model preheating device in an impulse test facility [31]. A small-scale arc-jet facility, OPG1, for integration, has been developed [32]. However, subcomponents and a vertical movement system for dual-facility operation in the expansion tube mode of the T6 Stalker tunnel appear to be still under development. As of now, no further advancements or results have been reported. However, there are a few obstacles that must be overcome to produce meaningful experimental results. First, the temperature of the model cannot be kept constant, as compared to using an electrical heating device. After preheating, it is difficult to experiment with the exact target temperature because the model is cooled by radiative heat fluxes from the surface while moving the model to perform the shock tunnel experiment. Second, there is a need for the development of measurement techniques that are suitable for high-temperature environments. Although the use of optical measurement methods far from the model is anticipated, it is important to note that this approach will have its own set of limitations. For instance, when using infrared thermography (IRT) technology for surface temperature measurement, achieving precise readings can be challenging due to the changing emissivity of the model surface during the preheating session. Lastly, aerodynamic phenomena such as ablation, variations in roughness, and the effect of wall-to-total temperature ratio are integrated and reflected in the experimental results. Therefore, it is imperative to develop techniques that can isolate these factors for a more detailed analysis.

This paper describes each part of the combined hypersonic test facility (the shock tunnel, arc jet tunnel, and horizontal model transport system), operating procedures, and test results. This equipment is structurally simple and economical to manufacture and operate on a small scale. Therefore, it is suitable at the university level. The concept of this equipment was patented in 2021 in South Korea [33], and its development history and related experimental results were first presented in 2022 [34]. This new type of combined facility covers various aerothermodynamic phenomena of testing conditions, including the surface temperature, surface roughness, and ablation effects, and represents a significant advancement over existing test facilities.

## Development of combined hypersonic test facility, K4

**Fig 3:**
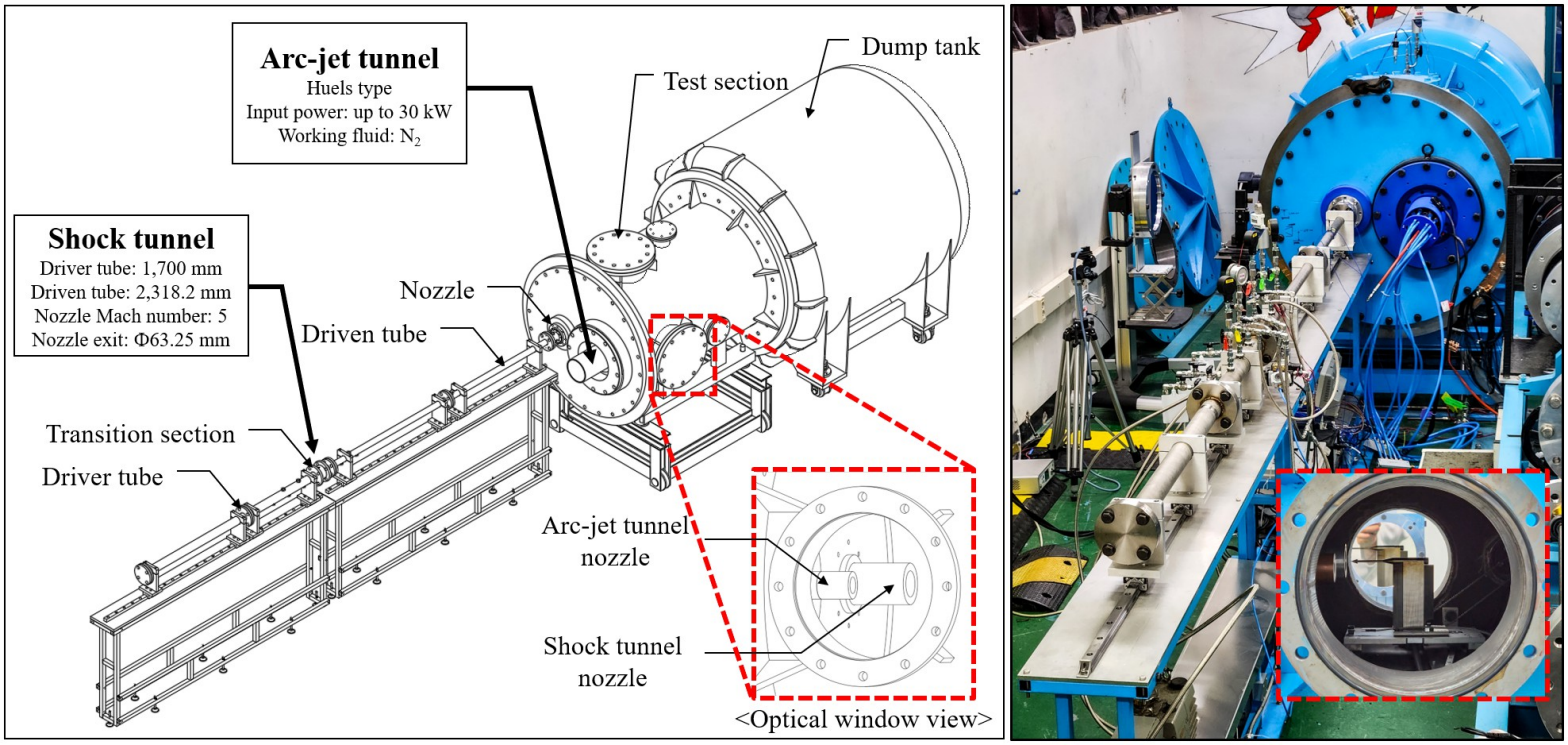
Schematic of arc-jet tunnel and shock tunnel combined facility (K4).

Fig 3 shows a schematic of the arc-jet and shock tunnels combined with the hypersonic test facility. This facility consists of an arc-jet tunnel, shock tunnel, model transport system, concurrently used test section, and dump tank. The detailed dimensions, component specifications, and piping and instrumentation diagram (P&ID) of each part are presented in the following sections.

Generally, a shock tunnel consists of a driver tube (high pressure), driven tube (low pressure), nozzle, and test section. An arc-jet tunnel consists of an arc heater (plasma torch), a nozzle, and a test section. The two test facilities were combined into a single facility by sharing a test section.

**Fig 4:**
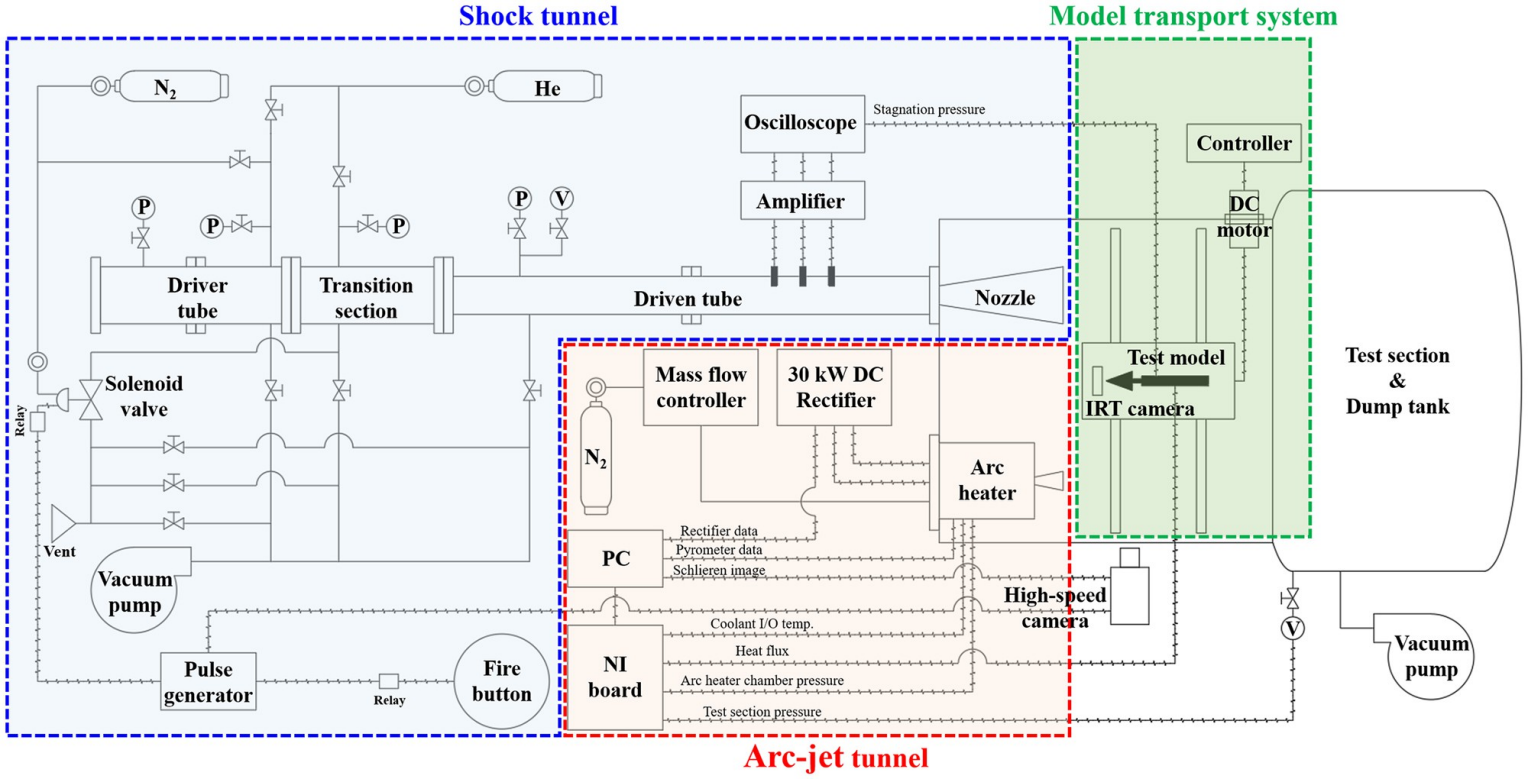
Piping and instrumentation diagram (P&ID) of K4.

Fig 4 shows the P&ID of the K4 system. The area highlighted in red illustrates the setup for the arc-jet tunnel operation, consisting of a nitrogen tank, mass flow controller (MFC), 30 kW DC rectifier, arc heater, and nozzle. The system also comprised a National Instruments Data Acquisition (DAQ) board and a personal computer (PC) for data acquisition. The area marked in blue was setup for shock tunnel operation. The setup consisted of a driver tube, driven tube, nozzle, helium tank, nitrogen tank, vacuum pump, solenoid valve, pulse generator, and oscilloscope. The green area represents the model transport system consisting of a DC motor and its controller.

### Development of arc-jet tunnel part

**Fig 5:**
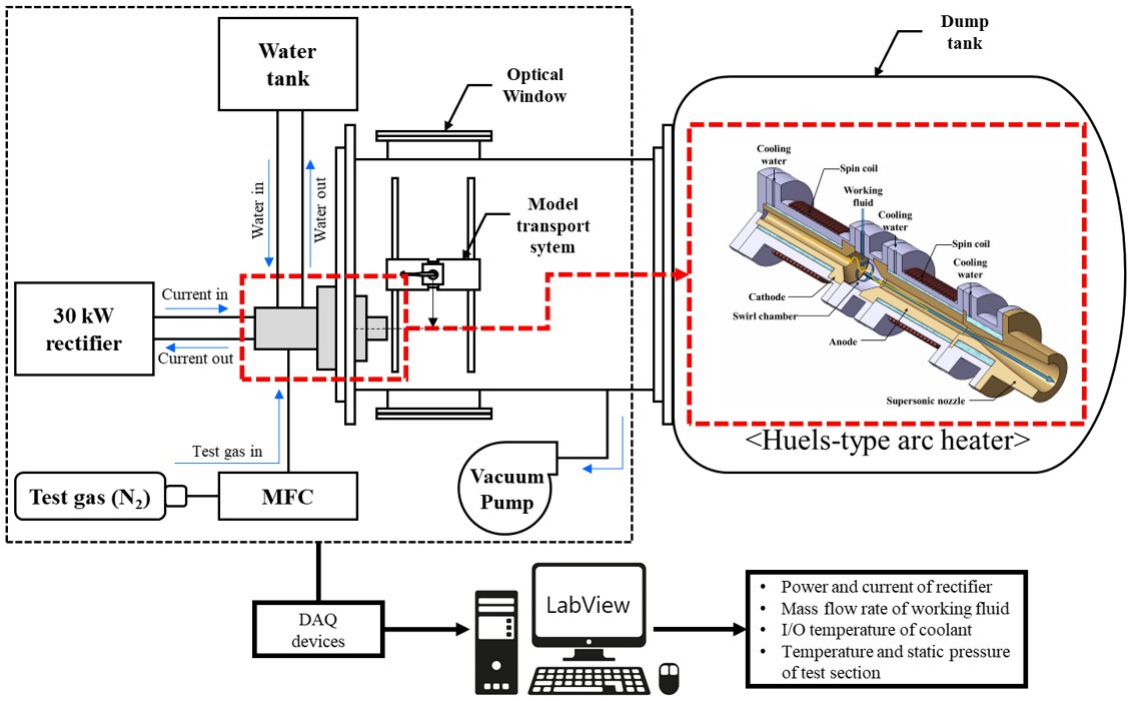
Schematic of Huels-type arc heater in K4.

Arc-jet tunnels are used to test the performance and durability of materials, structures, and other components under extreme conditions. The test gas, heated by the arc discharge between the electrodes, was accelerated into the test section using a supersonic converging–diverging nozzle. Various arc heaters (plasma torches) are used for constructing arc-jet tunnels, depending on the required ranges of the total pressure and enthalpy. These are the Huels, segmented, constricted, and hybrid types. The Huels and segmented constrictor types are widely used in aerospace engineering research because of the advantage of supplying a large amount of high-enthalpy flow under high total pressure conditions [35]. The Huels-type arc heater has a relatively simple structure in which the hollow-type cathode and anode are separated by a swirl chamber. An arc column was generated at the center of the electrodes, and the axial-vortex stabilization method was applied to stabilize the arc by the pressure distribution of the vortex flow induced by the tangentially injected working gas (N_2_ or air) in the swirl chamber. In addition, the magnetically stabilized method, in which the electrode is wrapped with a spin coil, was applied. This method is used to prevent arc root propagation to the rear plug and as a supplementary stabilization technique in addition to the aerodynamic stabilization method [36]. Fig 5 shows a schematic of the Huels-type arc-jet tunnel employed to construct K4. A 30 kW-class Huels-type arc heater used in the arc-jet tunnel was manufactured by Vitzro Nextech. The energy source for this system was a 30 kW DC rectifier, and the arc-jet tunnel was equipped with a water cooling system to mitigate thermal loads inside the arc heater and nozzle. It also comprised a working fluid (N_2_ or air) tank and an MFC to control the flow rate of the working fluid. The working fluid was introduced into the arc heater through the MFC, and the rectifier was turned on to generate high-enthalpy flow. When the flow was stabilized at the nozzle exit, the test model was moved from the center of the test section to the arc-jet nozzle using the model transport system to expose it to a high-enthalpy flow [33].

### Performance validation of arc-jet tunnel part

**Fig 6:**
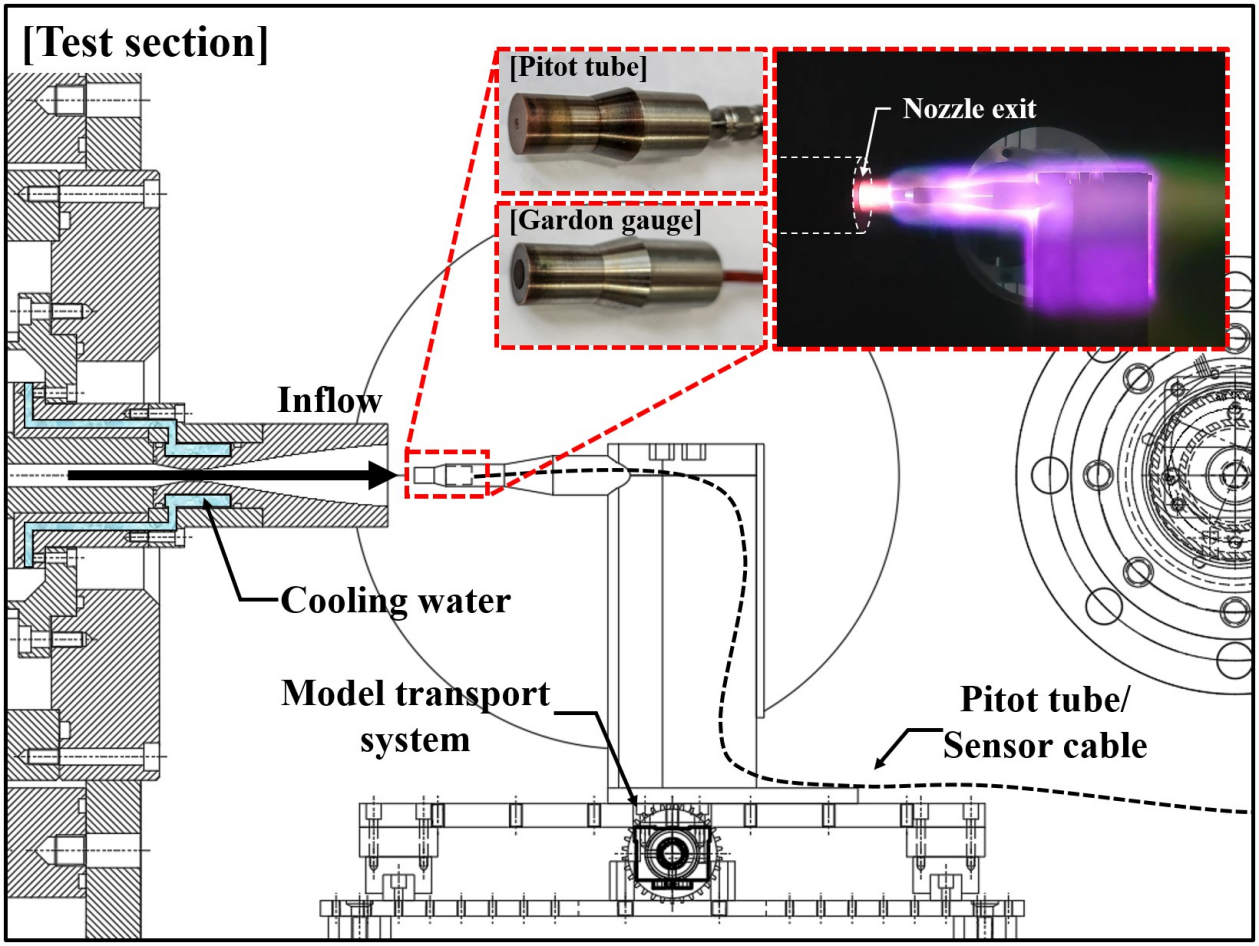
Schematic of performance validation experiments setup for arc-jet tunnel part.

Fig 6 shows a schematic of the performance validation experiments setup for the arc-jet tunnel. In the flow diagnosis test, the pitot pressure and heat flux were measured using a pitot tube and Gardon gauge, respectively, in the center of a model with the same flat-faced shape and a diameter of 10 mm. The pitot tube was constructed using a 1/16” stainless steel tube and connected to a pressure transducer outside the test section. The Vatell TG1000–0 model was used as the Gardon gauge to measure the heat flux at the stagnation point.

Table 1 lists the operating conditions used for the arc-jet flow diagnosis experiments. All experiments were performed under these conditions. Nitrogen with a mass flow rate of 1.03 g/s was used as the working fluid, and the rectifier was operated at 12.5 kW and 85 A.

**Table 1:** Operating conditions of arc-jet tunnel.

Parameters	Unit	Values
Working fluid(N_2_)	[g/s]	1.03
Power	[kW]	12.5
Current	[A]	85

**Fig 7:**
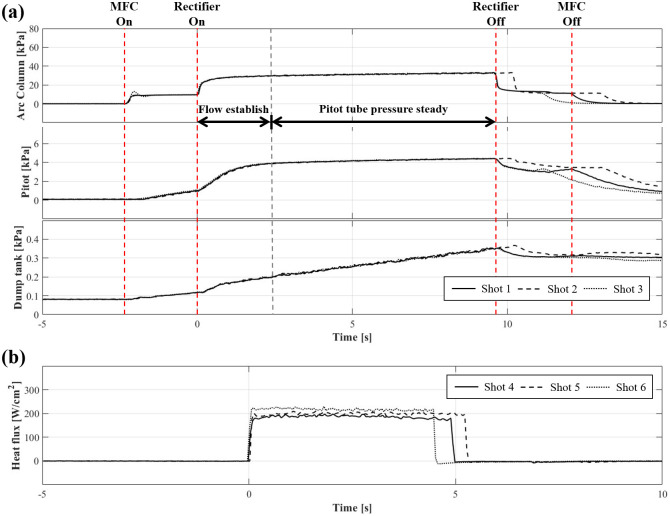
Test results of performance validation for arc-jet tunnel part. (a) Pressure measurements; (b) heat flux measurements.

Fig 7(a) shows the results of the simultaneous measurements of the pitot tube pressure, arc column (arc-heater inner chamber) pressure, and dump tank static pressure. The time was indicated based on when the rectifier was turned on. Considering the flow establishment time, the MFC was turned on at approximately —3 s, and the arc-jet tunnel was operated for approximately 10 s. The flow stabilized in the arc heater for approximately 3 s, and relatively steady pressure was maintained until the rectifier was turned off. During the test period, the arc column pressure was 32.6±0.08 kPa, and the measured pitot pressure was 4.39±0.005 kPa. The static pressure of the dump tank did not reach a steady state and tended to increase linearly owing to an insufficient effective pumping speed. Currently, during the arc-jet tunnel is operated for a duration exceeding 10 seconds, the static pressure of the test section rises, causing the nozzle exit flow to over-expand, and the generated oblique shock affects the reduction of the test rhombus. To address this issue, vacuum pumping tubes with larger diameters and additional vacuum pumps will be employed to maintain the dump tank static pressure at 250 Pa, similar to the nozzle exit pressure, to ensure flow stability to heat the test model to a higher temperature. Fig 7(b) shows the measured heat flux values for the stagnation point under the same operating conditions; the nominal value was 1.99±0.03 MW/m^2^.

### Development of shock tunnel part

**Fig 8:**
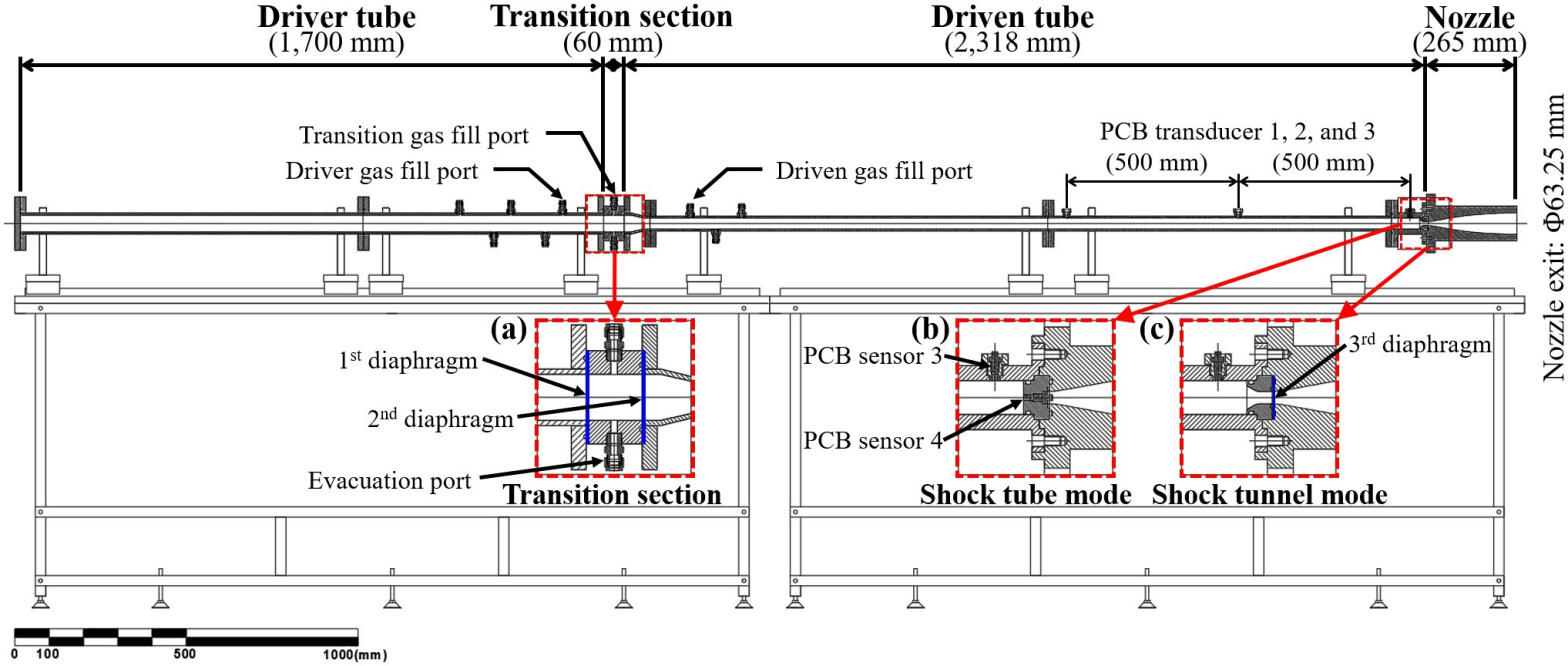
Schematic of shock tunnel part. (a) Transition section; (b) shock tube mode; (c) shock tunnel mode.

A new shock tunnel was developed for the combined hypersonic test facility. The shock tunnel consisted of a driver tube, transition section, driven tube, and M5 converging–diverging contour nozzle. The nozzle was installed at the end of the driven tube. The nozzle throat and exit diameters were 12 and 63.05 mm, respectively. A schematic of the shock tunnel and the detailed dimensions of each component are shown in Fig 7. The driver tube had a larger diameter than the driven tube and was separated by a tapered cross-sectional area. The shock tunnel operated using the double-diaphragm method, in which the first and second diaphragms on both sides of the transition section physically separated the driver and driven gases. When using the single-diaphragm method, consistent test conditions are difficult to achieve because the pressure required to rupture the diaphragm may vary, depending on the state of the diaphragm [37, 38]. This variability can negatively affect the repeatability of the experiments. Therefore, a new shock tunnel was designed using the double-diaphragm method. This method ruptures the diaphragm based on the pressure difference between the driver tube and the transition section, which occurs when the gas is momentarily discharged through the evacuation port in the transition section, as shown in Fig 8(a). The driver tube was filled with the driver gas to the target pressure (pressure fill condition), and the transition section was filled to a pressure less than the critical pressure of the diaphragm for its rupture. During the pressurization of the driver tube and transition section, it is important to carefully select the pressure fill conditions and the material, and thickness of the diaphragm to prevent it from bursting beyond the limits of both the first (located between the driver tube and transition section) and secondary diaphragm (located between the transition section and driven tube) diaphragms.

The shock tunnel was manufactured using a standard stainless steel (STS304) seamless pipe for economic manufacturing. 50A schedule 80 pipes, each with an outer diameter of 60.5 mm and an inner diameter of 49.5 mm, were used for the driver tube. 32A schedule 80 pipes, each with an outer diameter of 42.7 mm and an inner diameter of 32.9 mm, were used for the driven tube. At a temperature of approximately 320 K, the allowable working pressures for Schedule 80 pipes with diameters of 32A and 50A are 30.9 MPa and 24.1 MPa, respectively. These pressures are suitable for the fabrication of small-scale shock tunnels.

This facility could operate in the shock tube and shock tunnel modes by installing a slug-type plug between the end wall of the driven tube and the nozzle (Fig 8(b) and 8(c)). In the shock tube mode, a piezoelectric pressure (PCB) transducer was installed to measure the pressure at the end wall of the core flow inside the tube. In the shock tunnel mode, the slug-type plug was replaced by a nozzle throat with a third diaphragm. The driven tube was equipped with three PCB transducers as the flush-mounted type to determine the wall static pressure and shockwave propagation speed. Each sensor (PCB 1, 2, and 3) was installed at spaced intervals of 500 mm.

### Performance validation of shock tunnel part

The wall static and reservoir pressures were measured in the shock tube mode to validate the performance of the shock tunnel part, and the wall static and pitot tube pressures were measured in the shock tunnel mode. Pressure measurements using piezoelectric transducers have been widely used for flow diagnosis in shock tunnels [39–42]. In this section, the shock propagation speed and reservoir pressure inside the shock tube were measured using the sensors manufactured by PCB Piezotronics, and the performance of the equipment was verified through comparison with the theoretical values.

The flow characteristics inside the shock tube depend on the initial pressure filling conditions in the driver tube (driver gas, state 4) and driven tube (test gas, state 1). A shock wave called an incident shock was formed by rupturing the first and second diaphragms. It passed through the test gas and propagated downstream of the driven tube. Simultaneously, the expansion waves propagated in the direction opposite to that of the driver tube. The shock wave increased the temperature and pressure of the driven gas (state 2), and the expansion waves decreased the pressure and temperature of the driver gas (state 3). When the incident shock was reflected from the end wall of the driven tube and propagated to the opposite side (reflected shock), it increased the pressure and temperature of the driven gas (state 5). The steady test time required to maintain a homogeneous reservoir pressure is reached when the reflected shock and contact surface of the test and driver gases interact. The properties of each state can be calculated using the relationship between the initial pressure filling condition and the shock wave parameters (shock tube equation) [43, 44]. All experiments were performed under the test conditions listed in Table 2. This condition was also calculated theoretically.

**Table 2:** Calculated test conditions of shock tunnel part.

State	Gas	Pressure [kPa]	Temperature [K]	Speed of sound [m/s]
State 1	Air	32.0	290	341.62
State 2	Air	313.15	752.1	550.16
State 4	Helium	1,267.6	290	1,002.1
State 5	Air	1,530.8	1,322.1	729.43
Nozzle exit	Air	2.893	220.35	297.79

High-purity helium was used to fill the driver tube (state 4) and transition section at 1,270 and 600 kPa, respectively. The driven gas (state 1) was filled with dry air in the driven tube at 32.0 kPa. Polyethylene sheets, each with a thickness of 100 μm, were used as the first and second diaphragms, and a 40-μm-thick polyethylene sheet was used as the third diaphragm to sustain these conditions. Preliminary experiments confirmed that the first and second diaphragms ruptured at a pressure difference of 989 kPa. Under these conditions, the calculated speed of the incident shock wave was 997.539 m/s, which was converted to a Mach number of 2.92. The test flow pressure expanded by the M5 nozzle in state 5 (reservoir gas) was 2.893 kPa at 220.354 K, corresponding to a pressure altitude of approximately 24 km.

**Fig 9:**
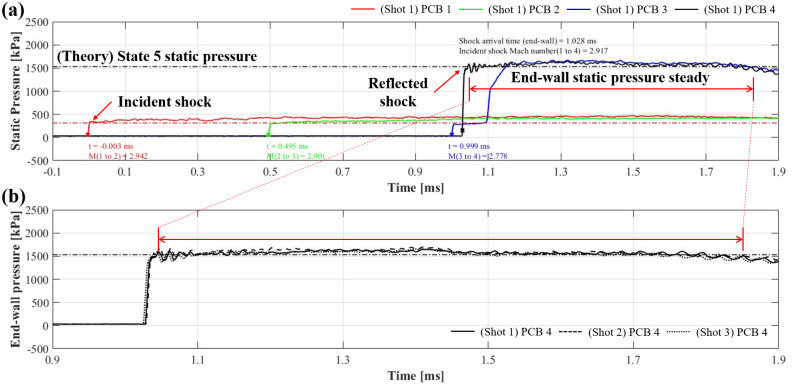
Shock tube test results. (a) Wall static pressures; (b) reservoir pressures.

Fig 9 shows the performance validation test results for wall static pressure measurements of the shock tube using the PCB transducers installed in the driven tube. All four PCB transducers belonged to the 111A26 series and had sensitivity values of 1.400, 1.475, 1.407, and 1.420 mV/kPa. Fig 9(a) shows the wall static pressures measured using PCB transducers 1, 2, and 3, and the reservoir pressures measured with PCB transducer 4 for shot 7. The reservoir pressure obtained during the experiment was 1,580±1.3 kPa, consistent with the theoretical value. In addition, the pressure of each PCB transducer was assumed to exceed 40 kPa (initial pressure: 32 kPa) as the shock arrival time to calculate the Mach number of the incident shock. The distance from PCB 1 to 4 was 1.028 m, and the duration of the incident shock passing through PCB 1 to 4 was 1.031 ms (ranging from —0.003 to 1.028 ms). Therefore, the calculated incident shock speed and Mach number at 290 K were 997.090 m/s and 2.917, respectively. Fig 9(b) confirms the repeatability by comparing the measurement results of PCB 4 for shots 7, 8, and 9. The steady-state was proven to be approximately 750 μs by selecting a time interval where the static pressure slope did not change within the range from the theoretical value of reservoir pressure ±10% (a gray area in Fig 9).

A nozzle flow diagnosis was conducted via pitot pressure measurement experiments to verify the performance of the shock tunnel. The stagnation pressure obtained from the pitot tube test can be used to determine the Mach number for the performance validation of the shock tunnel [45].

**Fig 10:**
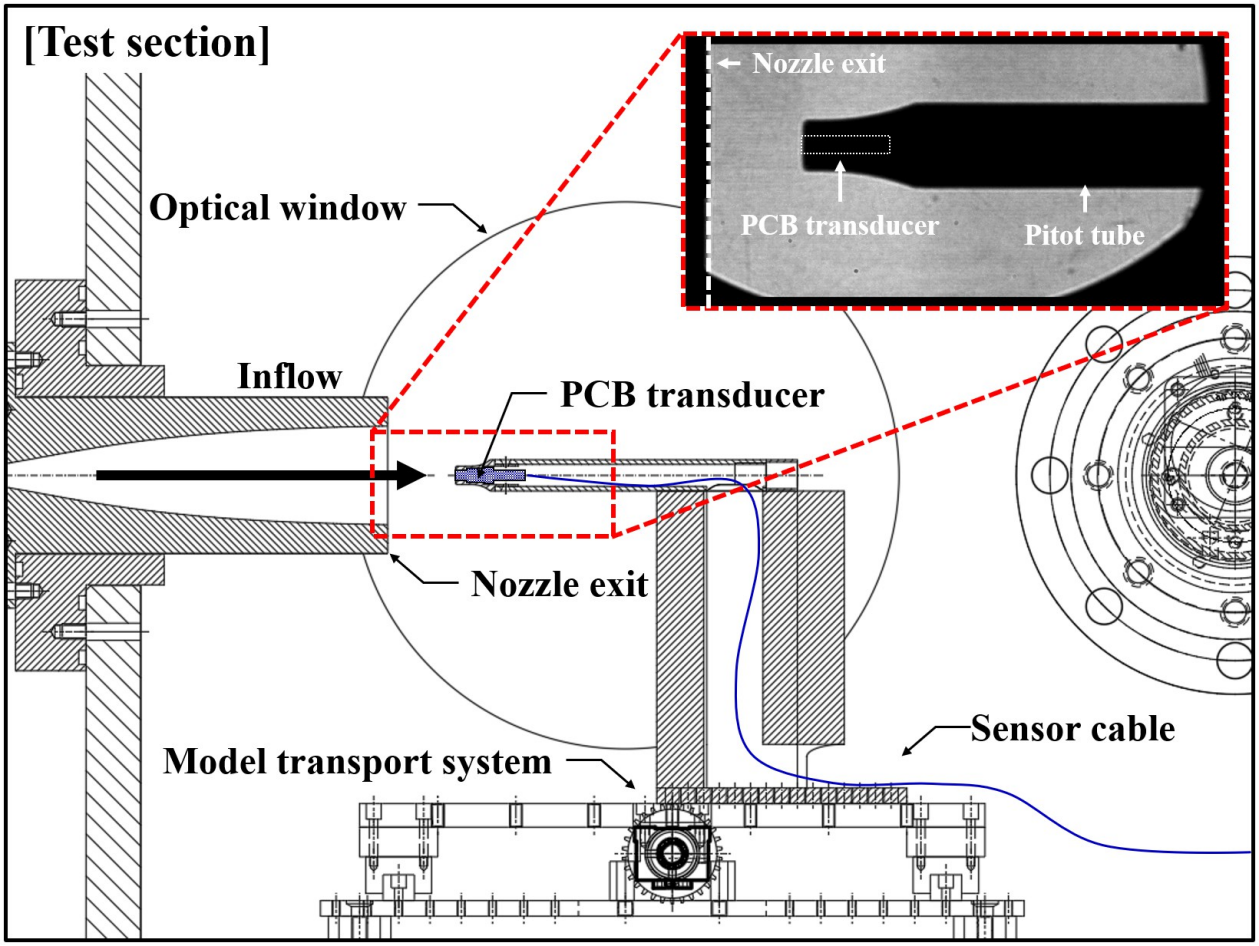
Schematic of performance validation experiments setup for shock tunnel part.

Fig 10 shows a schematic of the experimental setup for the pitot pressure measurements. The PCB transducer was installed at the center of the test model, which was PCB 4 (model: 111A26; sensitivity: 1.420 mV/kPa), and used in the shock tube mode to measure the pitot pressure. The conditions listed in Table 2 were applied. A Z-type shadowgraph was plotted using a high-speed camera to visualize the flow during the test.

**Fig 11:**
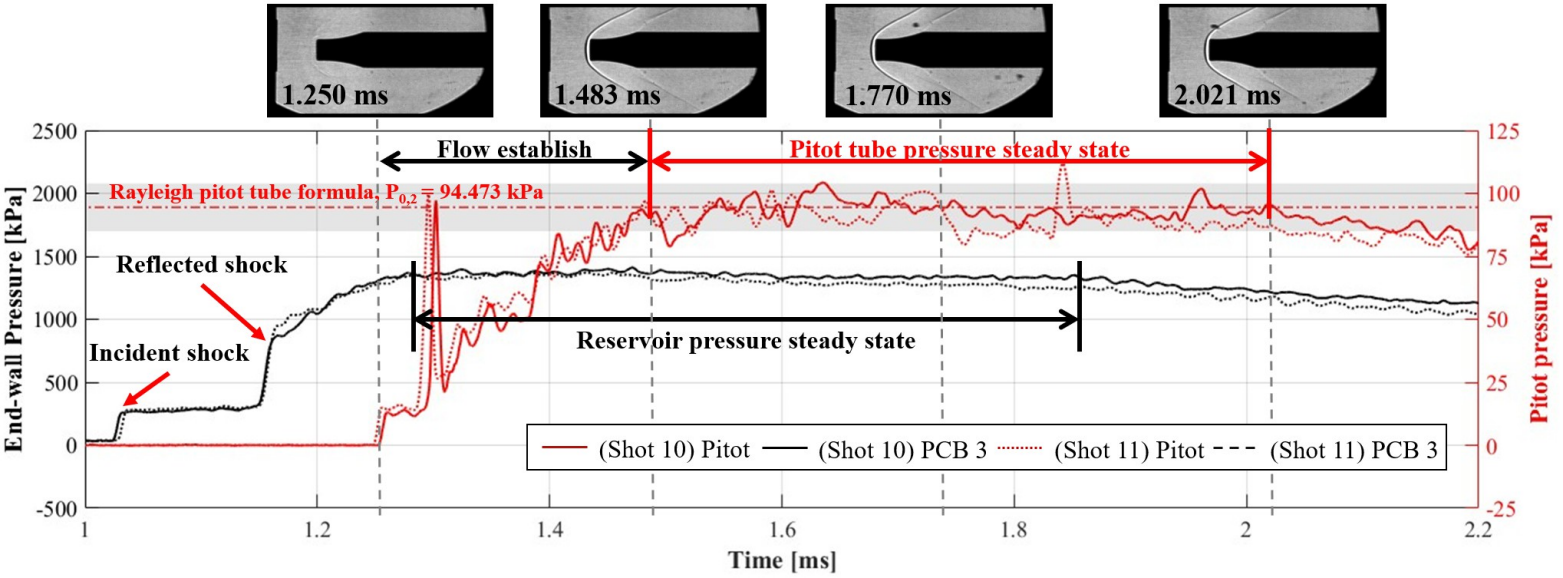
Result of the wall static and pitot pressure measurements in shock tunnel mode.

The pitot pressure measurement results are presented in Fig 11. When the reflected shock arrived, the third diaphragm installed at the nozzle throat ruptured, and the reservoir gas (state 5) expanded to the nozzle exit. A steady flow was obtained in the test section during the test time, excluding the flow establishment time at the nozzle. When the reservoir pressure (*P*_0_) was 1530.775 kPa, the pressure (*P*_1_) of the flow expanding through the M5 nozzle was 2.893 kPa. The pitot pressure calculated using the Rayleigh pitot tube formula was 94.473 kPa (*P*_0,2_). The wall static pressure (PCB 3) and pitot pressure traced for shots 10 and 11 were compared with the results of the Rayleigh pitot tube formula. The pitot pressure obtained from the tests was 91.8±0.22 kPa. The theoretical and experimental results were in good agreement. Similar to the shock tube experiment, the test time for the shock tunnel mode was approximately 520 μs.

### Model transport system

**Fig 12:**
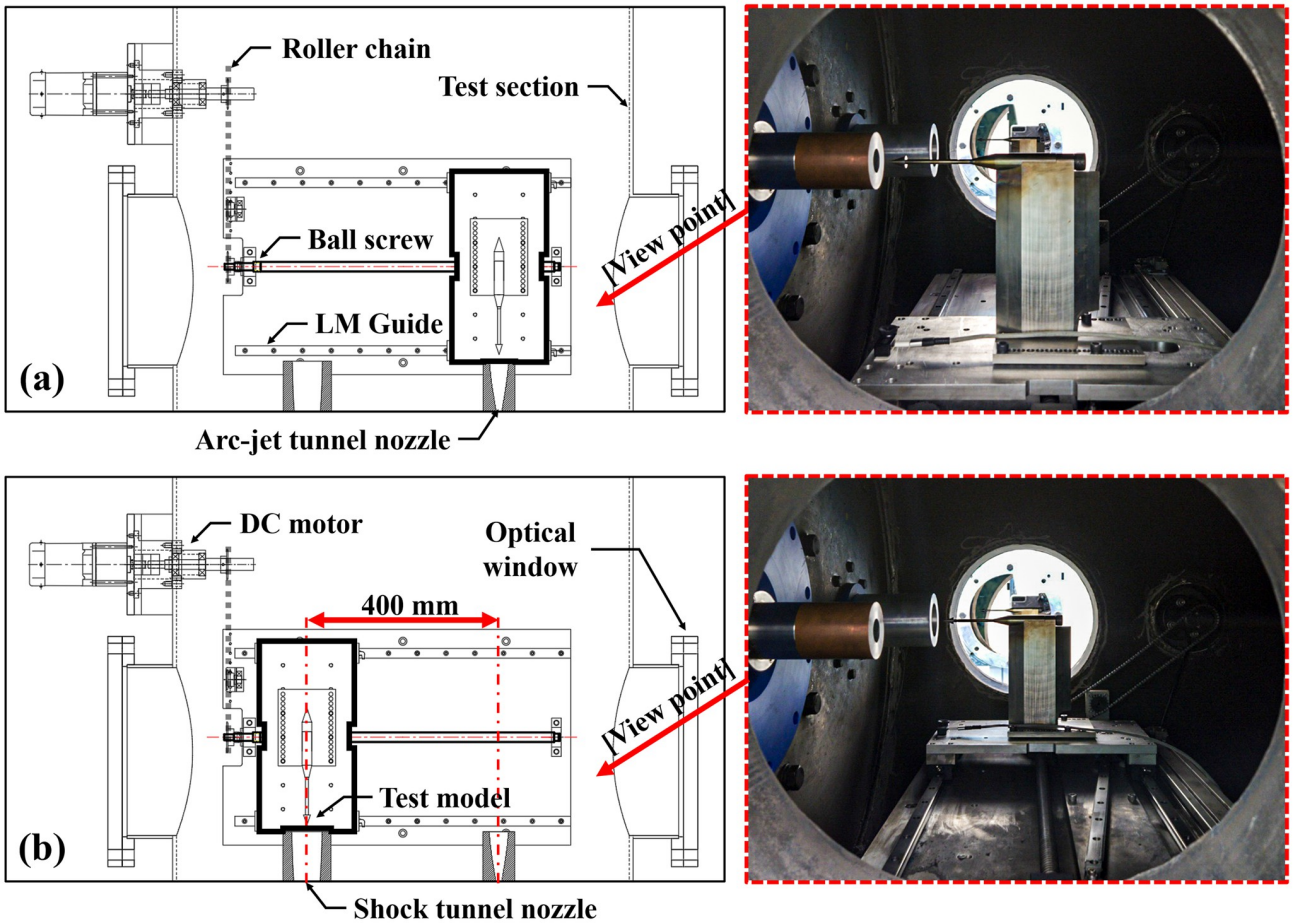
Schematic of model transport system. (a) Positioned at arc-jet tunnel; (b) positioned at shock tunnel.

Fig 12 shows a schematic of the model transport system and a photograph of the system in action, as observed from the optical window. Fig 12(a) shows the system positioned at the center of the arc-jet tunnel nozzle, and Fig 12(b) shows the system placed at the center of the shock tunnel nozzle. A chain-drive system was constructed using a DC motor, roller chain, ball screw, and LM guide. It was transported horizontally between the nozzle center of the arc-jet tunnel and the shock tunnel (400 mm). In the combined experiment, this transport system was centered in the test section before starting the arc-jet tunnel and was used to move the center of the arc-jet tunnel nozzle after the arc-jet flow had stabilized. It transferred the heated model to the shock tunnel after the operation of the arc-jet tunnel was completed for preheated testing with shock tunnel operation. Currently, this system moves at a speed of approximately 0.05 m/s and is manually controlled using a control panel.

## Experimental details of combined test

**Fig 13:**
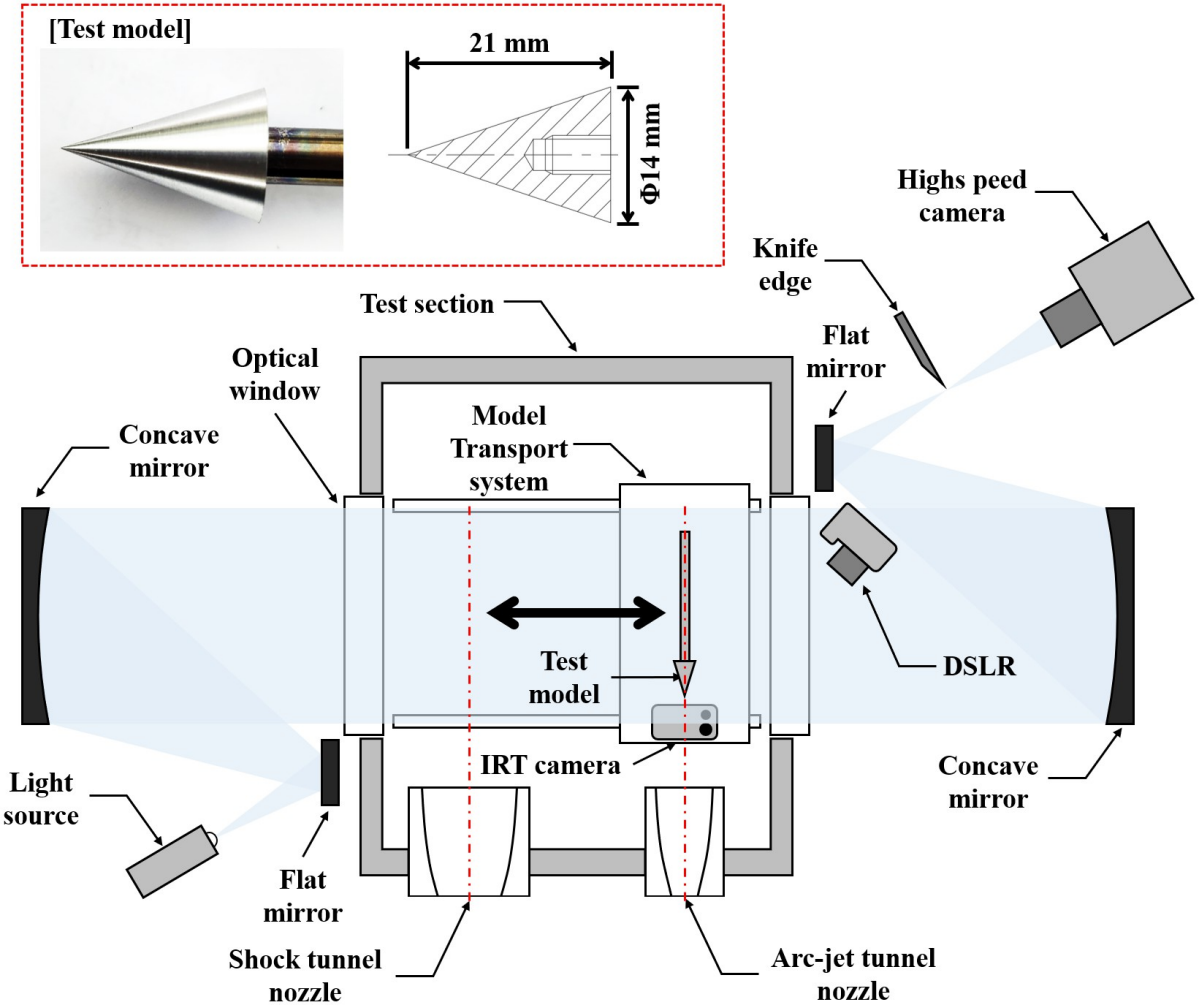
Schematic of experimental details of combined test with cone model.

Fig 13 shows a schematic of the experimental details of the combined test with a cone model. Fig 13 shows a schematic of the experimental details of the combined test with a cone model. The test model, constructed of AL6061, assumed a cone shape with a height of 21 mm and a diameter of 14 mm. An infrared thermography (IRT) camera (Seek’s Compact CW-AAA), which was a cable connected (USB-C type) to a smartphone for video display and savings, was installed under the test model in the test section during the integrated test period (resolution: 640×480; frame rate: 24 fps) to measure the surface temperature of the model. A Z-type Schlieren system comprising a high-speed camera (Photron’s Fastcam Mini UX100 with AF-S NIKKOR 24–70 mm lens), a light-emitting diode light source, two concave and flat mirrors, and a knife edge was used to visualize the shock tunnel flow (resolution: 1280×248; frame rate: 20,000 fps). A digital single-lens reflex camera (Canon’s EOS 600D with EFS 17–55 mm lens) was installed near the optical window to capture the operating video in the test section (resolution: 1920×1080; frame rate: 25 fps).

**Fig 14:**
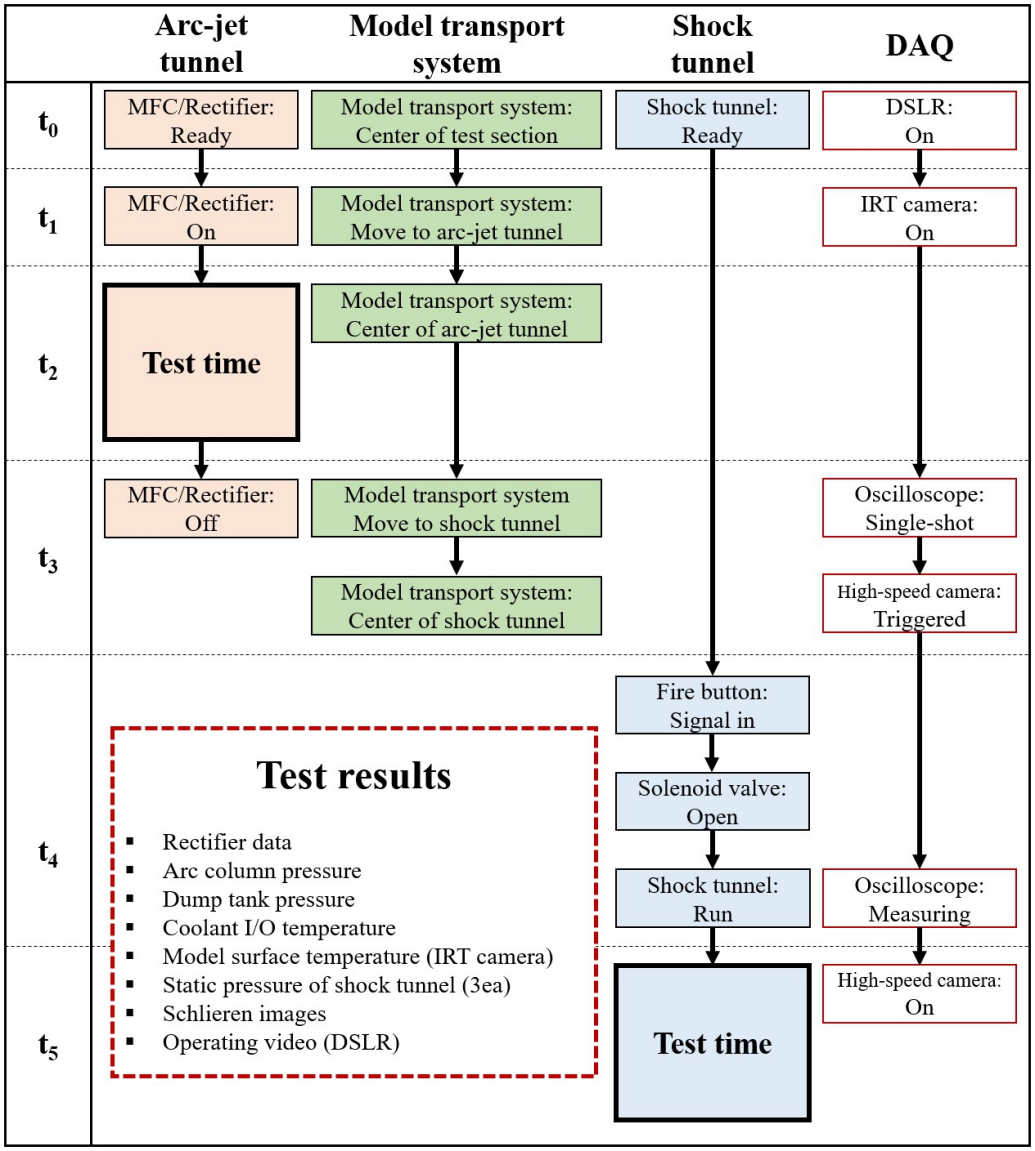
Operating procedure of combined test.

Fig 14 presents a concise overview of the chronological operating procedures for each part. Initially, at *t*_0_ (the ready state), the mass flow rate and injection time of the working fluid were set in the MFC, whereas the rectifier was configured with the current value in amperes. In addition, all measurement systems related to the arc-jet tunnel were turned on at this stage. The rectifier and PC exchange operating data (power and current) using serial communication (RS-485) and sensors for the arc column pressure, dump tank static pressure, and coolant temperature were connected to the DAQ board with the PC and measured during the combined test. The model transport system was positioned at the center of the test section. The driver tube, transition section, and driven tube of the shock tunnel were filled under pressure. The DSLR camera recorded the combined test operations.

At *t*_1_ (the first stage of the combined test), the MFC started supplying the working fluid, and the rectifier ignited the arc discharge in the arc heater. The high-enthalpy flow from the arc-jet tunnel reached the test section, while the fluid in the model transport system started flowing towards the center of the arc-jet tunnel. The IRT camera for the surface temperature measurements started recording.

During the *t*_2_ period (the arc-jet tunnel test time), the test model was exposed to a stable plasma flow using a model transport system that heated the test model to the target temperature.

At *t*_3_ (the shock tunnel test preparation stage), the arc-jet tunnel test was terminated, and the preheated hot-surface test model was transported to the center of the shock tunnel by the model transport system. The oscilloscope was connected to the PCB transducers, and a high-speed camera was set as the recording trigger.

At *t*_4_ (the shock tunnel operating stage), the signal from the fire button opened the solenoid valve on the evacuation port in the transition section. This caused ruptures in the first and second diaphragms and foamed the incident shock. When the incident shock arrived at PCB 1, the oscilloscope began recording the wall static pressures of PCB 1, 2, and 3.

Finally, at *t*_5_ (the shock tunnel test time), the high-speed camera captured videos of the flow around the preheated model throughout the test period.

After all the combined tests were completed, the experimental results were obtained (Fig 14).

## Results and discussion

**Fig 15:**
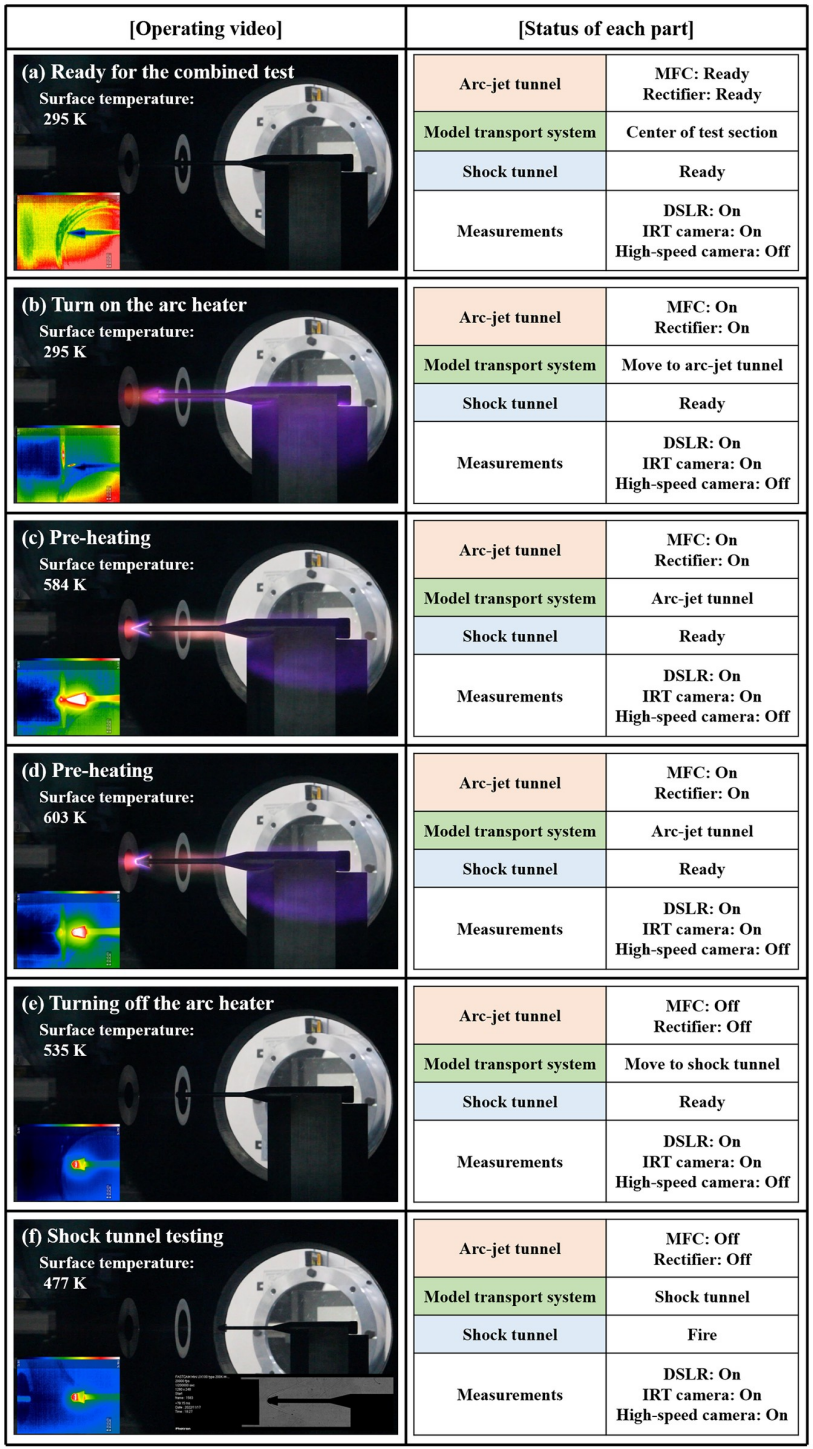
Operating images and status of each part during combined test.

In Fig 15, the operating video (DSLR), surface temperature (IRT camera), and Schlieren image (high-speed camera) are organized according to the operating procedure, and the status of each part at each moment is shown.

**Fig 16:**
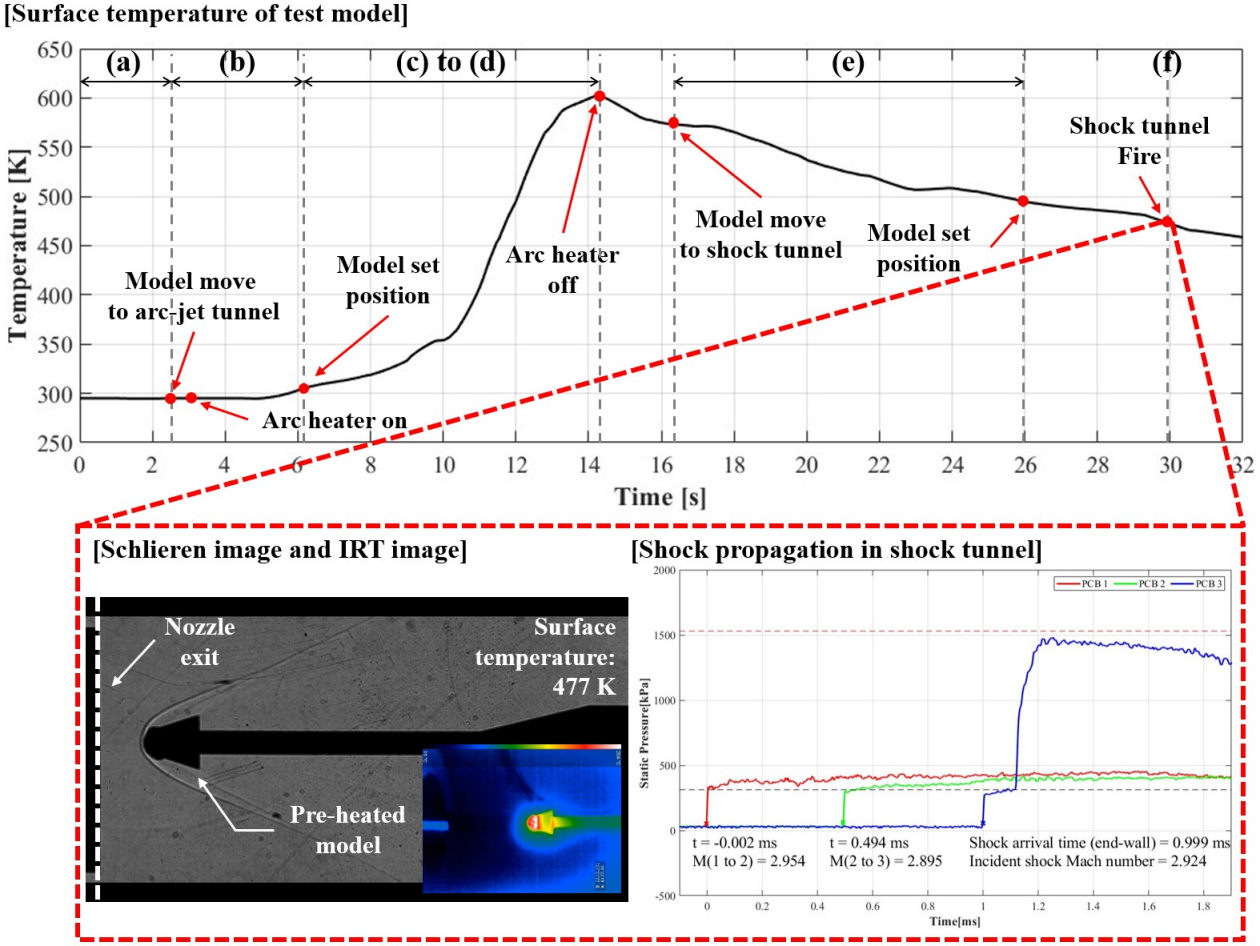
Results of combined test with cone models.

Fig 16 shows the test results of this integrated experiment as variations in the model surface temperature with time. Time stamps (a)–(f) in Fig 16 correspond to Fig 15(a)–15(f). Interval (a) represents the preparation time for the combined test stage (*t*_0_ = 2.5 s). Interval (b) represents the arc heater ignition stage (*t*_1_ = 3.0–6.1 s). Between periods (c) and (d), the test model was heated from 295 to 603 K. The duration of the heating phase was approximately 8.2 s (*t*_2_ = 6.1–14.3 s). During this phase, ablation was initiated in front of the model at 13 s. Interval (e) represents the state of turning off the arc heater; the test model was transported to the shock tunnel, and simultaneously, the shock tunnel was prepared for operations (from *t*_3_ = 16.3 to *t*_4_ = 25.96 s). Time stamp (f) represents the moment the shock tunnel experiment was finally performed using the hot-surface test model (*t*_5_ = 29.9 s). A shock tunnel test was conducted using the test model at a surface temperature of 477 K.

The surface temperature of the model was measured 24 times per second, and a moving average filter was used to compensate for the stopping phenomenon in its application, which appeared to have occurred during the communication process between the IRT camera and smartphone. The temperature measurements performed in this experiment using the IRT camera cannot determine the exact temperature because the emissivity value varies with the state of the model surface, which changes during heating; however, it can be confirmed whether the model is heated.

**Fig 17:**
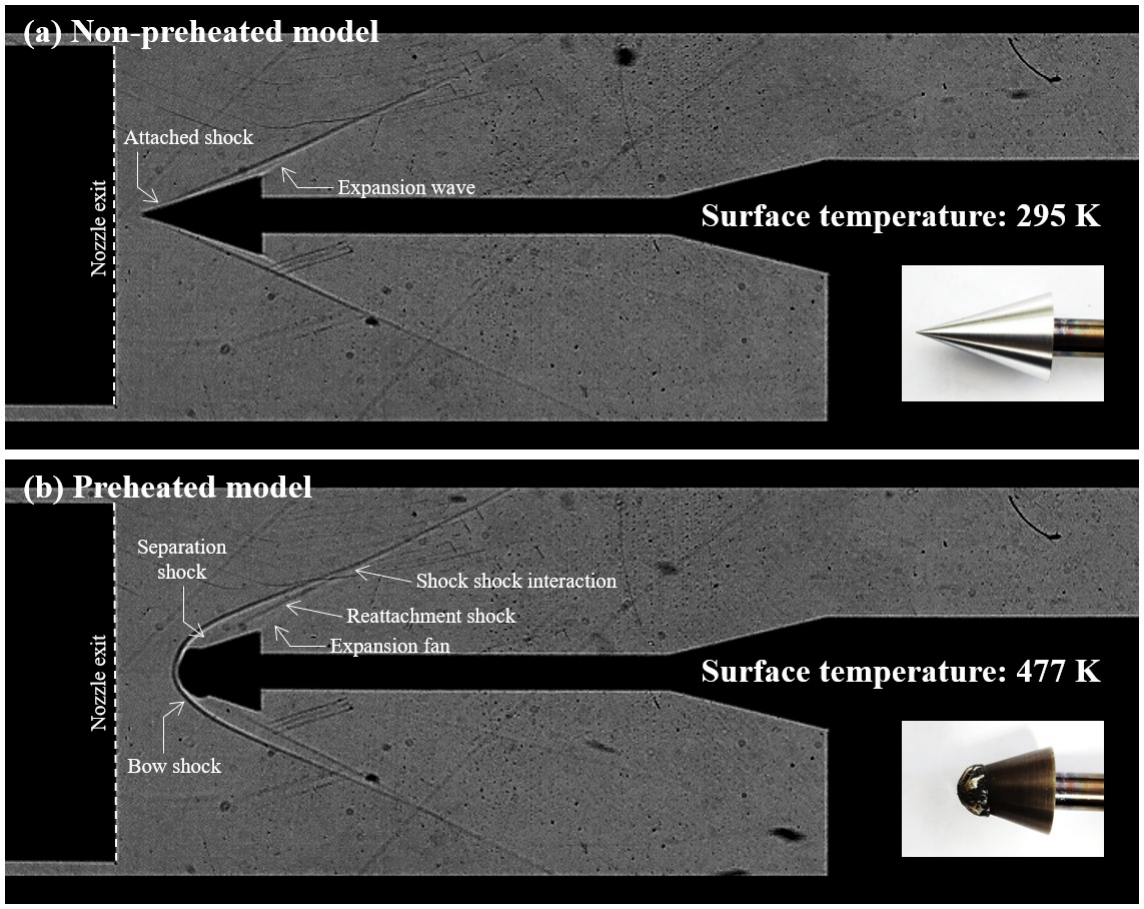
Fluid-structure of the non-preheated and preheated models. (a) Non-preheated model (295 K); (b) Preheated model (477 K).

Fig 17 presents a comparative analysis of the shock wave structure around the model at room temperature and the preheated model. In (a), a Schlieren image captured from an experiment performed solely in the shock tunnel at K4 before the combined test is depicted. Notably, attached shocks and an expansion wave are observed. (b) showcases a visualization image captured during the combined test. During the preheating session, ablation altered the model’s geometry, resembling a blunted double-cone. Consequently, the shock wave structure underwent significant changes. Attached shocks transformed into detached bow shocks, and phenomena such as separation shock, reattachment shock, and shock-shock interactions became observable. This observed phenomenon mirrors conditions anticipated at the leading edge of a practical hypersonic vehicle like a scramjet. The combined test using this cone model effectively verified the effects of ablation induced by preheating.

**Fig 18:**
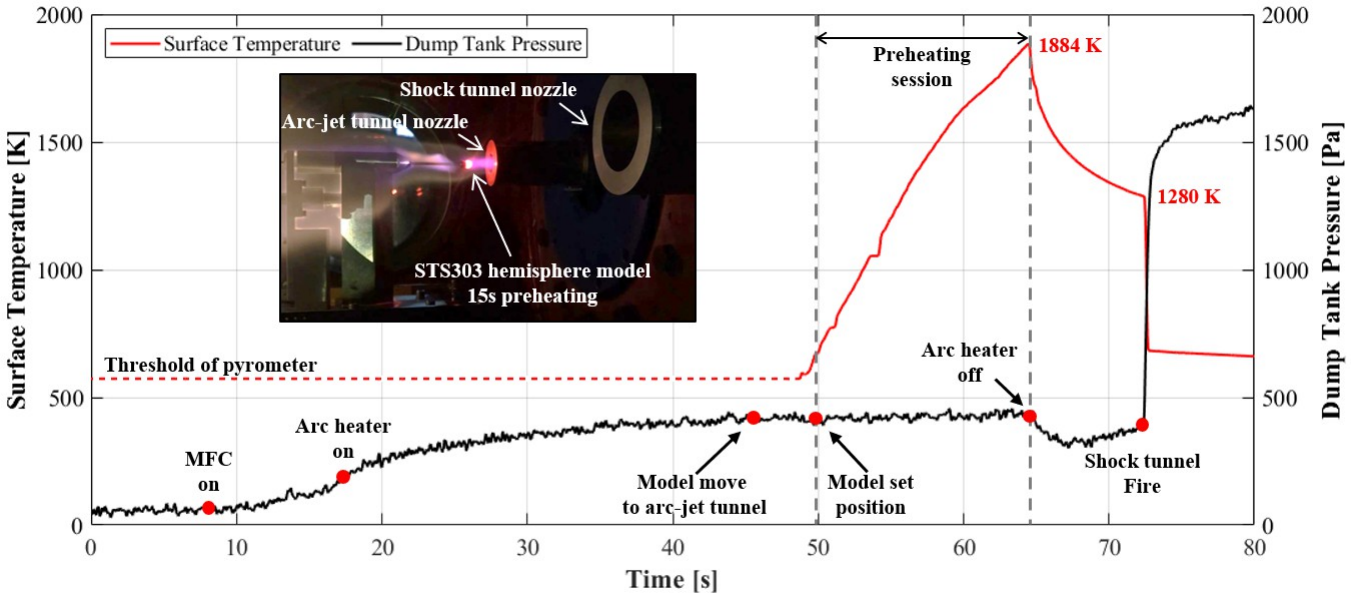
Surface temperature of hemisphere model and dump tank pressure during combined test.

Fig 18 illustrates the surface temperature of the model over time, recorded by the pyrometer (left y-axis), in conjunction with static pressure in the test section (right y-axis). The schematic of experimental details, featuring the hemisphere model and one-color pyrometer (AST’s A250+ FO PL, temperature range: 573 K to 2773 K and spectral range: 1.6 μm) The test model, characterized by a one-side hemispherical cylinder shape with a radius of 5 mm, height of 15 mm, and diameter of 10 mm, underwent examination in this experiment. A pyrometer, synchronized with the model supporter, measured the surface temperature at a frequency of 20 Hz throughout the entire duration, spanning from the preheating session to the shock tunnel experiment. The emissivity setting was established at 0.25, corresponding to the known emissivity of polished stainless steel. S1 Fig shows a schematic of the experimental details of the combined test with a cone model.

Commencing from a surface temperature of 573 K, the minimum measurement range of the pyrometer, specific operational milestones are marked with red circles on the static pressure data. Following a stabilization time of approximately 30 seconds subsequent to the operation of the MFC drive and arc heater ignition, the model was exposed to the arc-jet tunnel flow, experiencing a temperature rise to 1884 K during a preheating session lasting about 15 seconds. The model was then transported to the center of the shock tunnel, where the shock tunnel experiment was conducted with the surface temperature of the test model at 1280 K. SEM images captured at various magnifications are displayed in S2 Fig.

**Fig 19:**
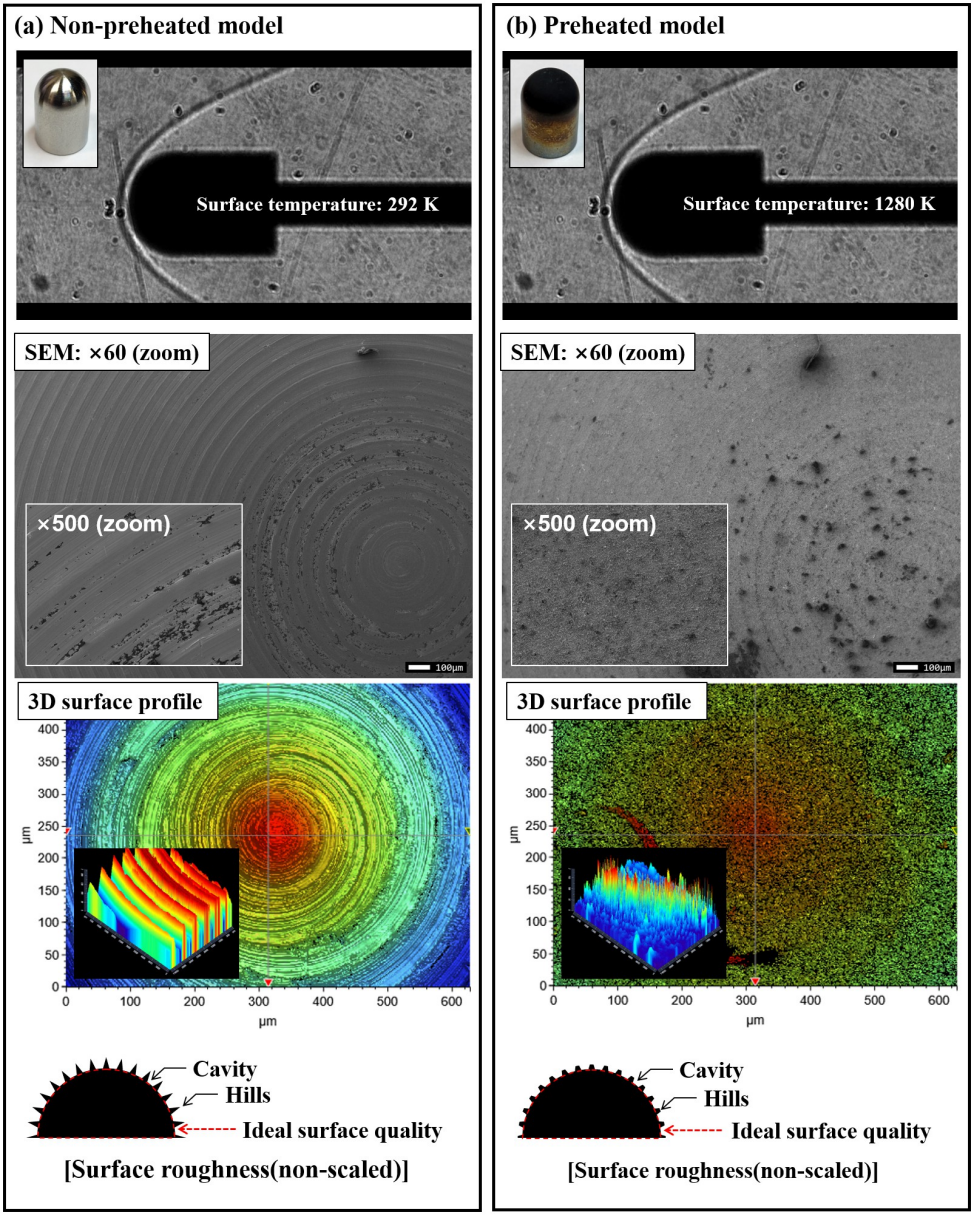
Results of combined test with hemisphere model.

Fig 19 presents a comparison between non-preheated and preheated hemisphere models. Flow visualization analysis did not reveal significant differences, attributed to optical system limitations such as light source intensity and high-speed camera lens magnification. To scrutinize model surface conditions before and after preheating, scanning electron microscopy (SEM) images and three-dimensional surface profile measurements via time-of-flight secondary ion mass spectrometry were utilized. In (a) of Fig 19, the surface of the model before heating is displayed, contrasting with (b) showcasing the surface after heating. Notably, the non-preheated model exhibits a distinct machining end surface, a result of the manufacturing process employing a milling machine. In contrast, the preheated model displays a reduced height of the cavity in the machining end.

Changes in shape due to ablation [46], alterations in surface roughness [47, 48], and variations in surface temperature [49] are well-established factors with substantial impacts on the aerodynamic performance of hypersonic vehicles. The morphological modifications resulting from these factors hold the potential to significantly influence aerodynamic characteristics. This underscores the critical importance of considering surface conditions when evaluating the aerodynamic consequences of preheating. A comprehensive understanding of how these elements interplay is crucial for optimizing the design and performance of hypersonic vehicles in diverse operational scenarios.

To dissect the individual impacts of aerothermodynamic phenomena on aerodynamic forces—specifically, the effects of surface temperature, roughness variations, and shape changes due to ablation—an experimental technique for drag force measurement will be devised and executed on the hypersonic combined facility K4. Anticipated outcomes include the ability to compare the drag forces of the preheated model and the cooled model (after preheating), providing insights into the thermal surface effects. Additionally, a comparison between the drag forces of the non-preheated model and the preheated model will be conducted, allowing for the assessment of ablation effects or roughness change effects based on the geometric characteristics of the model. This experimental approach will contribute valuable data for a nuanced understanding of the distinct contributions of these aerothermodynamic factors to overall aerodynamic performance.

Presently, surface temperature is measured using an IRT camera and a one-color pyrometer, which poses limitations in accuracy due to the varying emissivity of the model surface during the preheating session. To address this issue, a method will be implemented to correct for the uncertainty in temperature readings due to uncertainty in surface emissivity estimates from oxidized models [50]. Additionally, temperature correction procedures will be applied, considering the measurement angle of the pyrometer [51]. These advancements in measurement techniques aim to enhance the accuracy and reliability of surface temperature measurements, providing more precise data for the investigation of aerothermodynamic phenomena during preheating sessions.

## Conclusions

In this study, a new type of test facility was developed by combining a shock tunnel with a Huels-type arc-jet tunnel for a preheating apparatus into a single test section to overcome a limitation of impulse-type facilities, which is the inability to achieve temperature simulation of an actual hypersonic flight object surface owing to the short test duration. Moreover, an integrated test method using this test facility was proposed. A test model preheated using an arc-jet tunnel was transferred to the shock tunnel by the model transport system, and a shock tunnel test was performed continuously for aerothermodynamic studies. The effects of ablation-induced shape change and roughness change on the fluid-structure were investigated by combined experiments using two models with different materials and shapes. This equipment is suitable for university-level research facilities because of its small scale, simple structure, and high economic feasibility for manufacturing and operation. The experimental results obtained using this method highlight the potential and surprising capabilities of the combined hypersonic test facility. In addition, it not only demonstrates the successful integration of arc-jet tunnels and shock tunnels but overcomes the limitations of impulse-type facilities. The results obtained using this integrated test rig firmly establish its importance and validity, making it a promising tool for future research on aerothermodynamic phenomena. A process is underway to solve the weaknesses of the current equipment described above such as the fact that the static pressure of the dump tank is not maintained and the surface temperature of the test model is decreased owing to the slow speed of the model transport system. A force measurement experiment using a preheated model with an accelerometer is being conducted at this facility based on the test methods as a follow-up study. In the follow-up study, the outcomes of the non-preheated model, preheated model, and the subsequently cooled model will be compared to explore the integrated or separate effects of aerothermodynamic phenomena on aerodynamic forces.

## Supporting information

S1 Table Hot experimental technique with electrical heating device in impulse-type facility. (DOCX)

S1 Fig Schematic of experimental details of combined test with hemisphere model. (TIF)

S2 Fig Scanning electron microscopy (SEM) of surface roughness. (a) Non-preheated hemisphere model (STS303); (b) Preheated hemisphere model (STS303).
(TIF)
