# Supplementary material for: Development of combined hypersonic test facility for aerothermodynamic testing
Source: PLoS One. 2024 Feb 14;19(2):e0298113. doi: 10.1371/journal.pone.0298113 (PMC10866511; doi:10.1371/journal.pone.0298113)
Supplement: S1 Table — (DOCX) [file pone.0298113.s001.docx]

S1 Table. Hot experimental technique with electrical heating device in impulse-type facility

| **Years** | **Institutions** | **Preheating methods** | **Surface temp. [K]** | **Facility** | **Test models** | **Ref.** |
| --- | --- | --- | --- | --- | --- | --- |
| 2000 | RWTH Aachen University | Electrical resistance heating | 700 | TH2 shock tunnel | Ramp model | [12] |
| 2004 | The University of Queensland | Electrical element (copper plate) | 500 | T4 shock tunnel | Scramjet intake model | [13] |
| 2006 | The University of Queensland | Ceramic plate with heating element | 700 | T4 shock tunnel | Intake model | [14] |
| 2006 | RWTH Aachen University | Resistance heating elements | 840 | TH2 shock tunnel | Ramp model | [15] |
| 2009 | RWTH Aachen University | Electrical heating elements | ≤ 1000 | TH2 shock tunnel | Intake model | [16] |
| 2011 | RWTH Aachen University | Electrical heating elements | ≤ 1000 | TH2 shock tunnel | Intake model | [17] |
| 2013 | The University of Queensland | Resistive heating | 2000 | X2 expansion tube | Half-cylinder model | [18] |
| 2014 | RWTH Aachen University | Electrical heating elements | ≤ 1000 | TH2 shock tunnel | Isolator model | [19] |
| 2015 | Air Force Institute of Technology | Resistive heating | ≤ 3280 | X2 expansion tube | Half-cylinder model | [20] |
| 2016 | The University of Queensland | Resistive heating | ≤ 2410 | X2 expansion tube | Half-cylinder model | [21] |
| 2016 | German Aerospace Center (DLR) | Electrical heating element | 800 | High Enthalpy Shock Tunnel Göttingen | Flat plate with deflectable flap | [22] |
| 2017 | The University of Queensland | Resistive heating | 3300 | X2 expansion tube | Half-cylinder model | [23] |
| 2017 | University of New South Wales | RCC heater | ≤ 1130 | TUSQ Ludwieg tunnel facility | Flat plate model | [24] |
| 2018 | The University of Queensland | Copper electorde | ≤ 3210 | X2 expansion tube | Slotted cylinder and graphite strip | [25] |
| 2018 | University of New South Wales | Graphite heater plate | 800 | T-ADFA shock tunnel | Heated tick model | [26] |
| 2019 | The University of Queensland | Copper electorde | ≤ 2900 | X2 expansion tube | Wedge model | [27] |
| 2021 | The University of Queensland | Copper electorde | ≤ 3000 | X2 expansion tube | Wedge model | [28] |
