# Supplementary figures and images for: Development of combined hypersonic test facility for aerothermodynamic testing
Source: PLoS One. 2024 Feb 14;19(2):e0298113. doi: 10.1371/journal.pone.0298113 (PMC10866511; doi:10.1371/journal.pone.0298113)

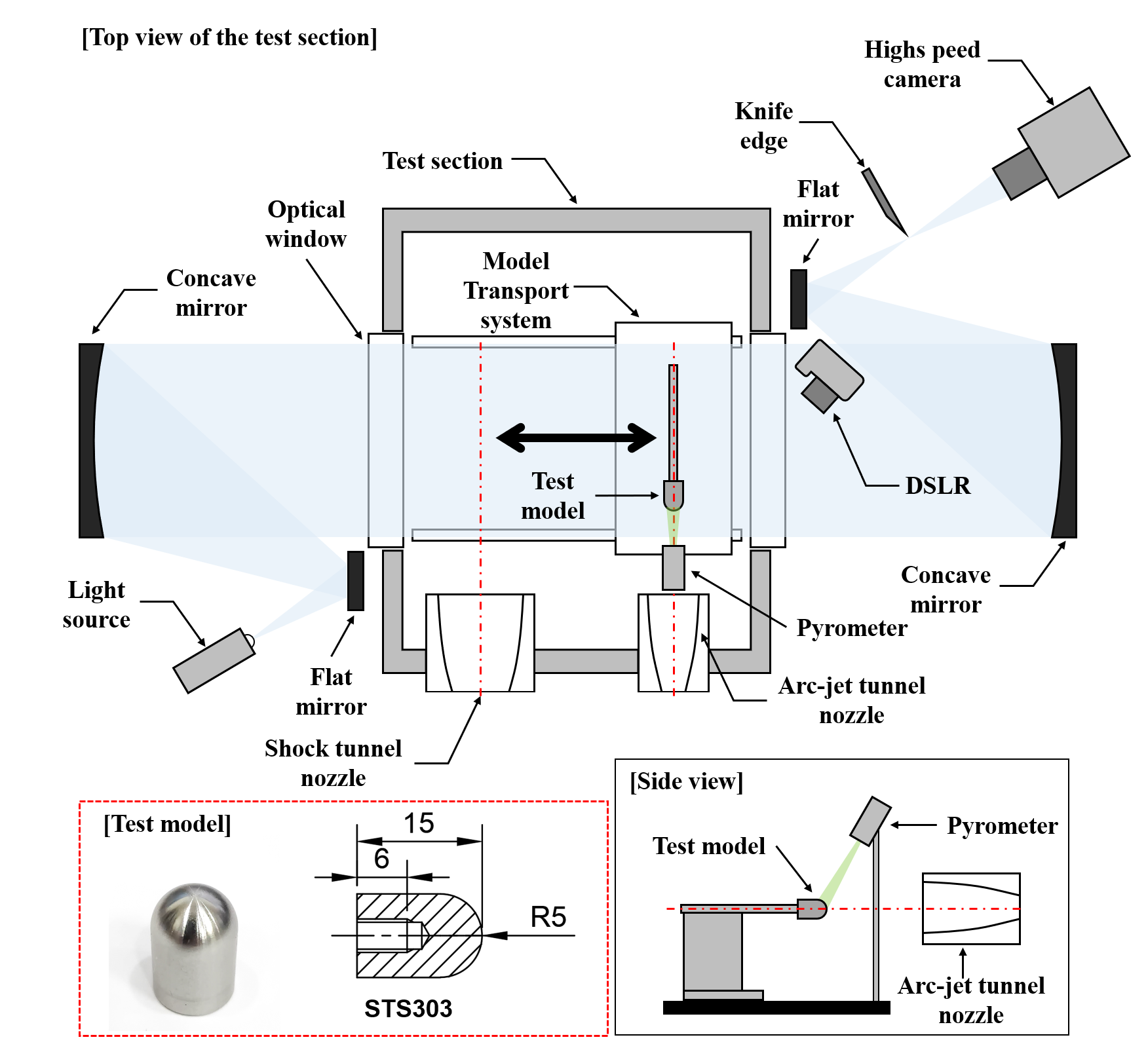

Supplement: S1 Fig — (TIF) [file pone.0298113.s002.tif]

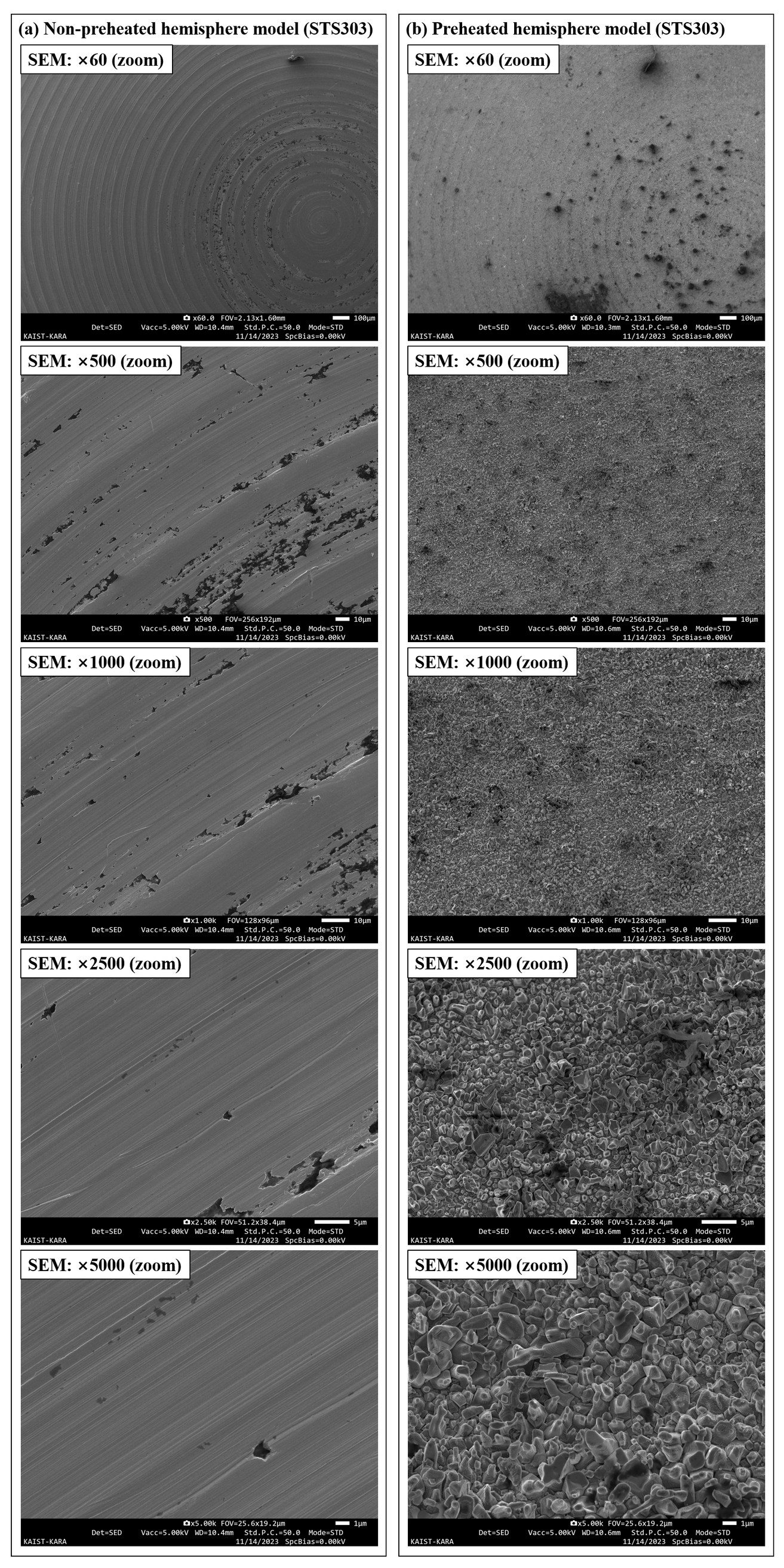

Supplement: S2 Fig — (a) Non-preheated hemisphere model (STS303); (b) Preheated hemisphere model (STS303). (TIF) [file pone.0298113.s003.tif]
